# Limonene and its metabolite perillyl alcohol inhibit Chlamydia trachomatis growth by altering host isoprenoid metabolism

**DOI:** 10.1007/s13659-026-00611-5

**Published:** 2026-04-13

**Authors:** Pilar Cebollada, Inés Reigada, Maarit Ylätalo, Candela Gerediaga, Víctor López, Leena Hanski

**Affiliations:** 1 Department of Pharmacy, Faculty of Health Sciences, Universidad San Jorge, 50830 Villanueva de Gállego, Saragossa Spain; 2 Drug Research Program, Division of Pharmaceutical Biosciences, Faculty of Pharmacy, University of Helsinki, 00014 Helsinki, Finland; 3 Instituto Agroalimentario de Aragón-IA2, CITA-Universidad de Zaragoza, 50013 Saragossa, Spain

**Keywords:** Intracellular bacteria, Protein prenylation, Membrane structural integrity, Perillyl alcohol, Monoterpen

## Abstract

The obligate intracellular bacterium *Chlamydia trachomatis* is the most common bacterial sexually transmitted infection globally, with approximately 131 million new cases each year. It contributes to widespread reproductive health issues, including infertility and chronic pelvic pain. The unique cell morphology and biphasic life cycle pose challenges to their effective eradication. Guided by the identification of essential oils (EOs) capable of suppressing *C. trachomatis* intracellular growth, this study evaluated the antichlamydial properties of the monoterpene limonene and its metabolites perillyl alcohol and perilic acid. The antichlamydial activity was assessed through qPCR-based quantification of bacterial genome copy numbers and immunofluorescence staining of chlamydial inclusions to monitor bacterial growth and infectious progeny production. Elementary body membrane integrity was assessed with viability PCR. *Citrus limon* essential oil exhibited dose-dependent inhibition of *C. trachomatis* growth, decreasing infectious progeny by over 90%. *Pinus sylvestris* EO displayed consistent but non-dose-dependent effects. Limonene, a major constituent of the EOs, exhibited significant suppression of chlamydial growth and progeny production, particularly for its R-enantiomer. While viability-PCR data indicated that the EOs and limonene affected EB membrane permeability, EB infectivity was not affected by the treatments. Perillyl alcohol suppressed chlamydial growth at a lower concentration (100 µg mL^−1^) than the parent compound limonene. The antichlamydial activity of both limonene and perillyl alcohol was suppressed when infected cultures were supplemented with farnesyl or geranylgeranyl, indicating that the antichlamydial mechanism of the two monoterpenes involves competitive inhibition of protein prenylation. Hence, targeting host protein prenylation may be an effective antichlamydial strategy.

**Graphical Abstract:**

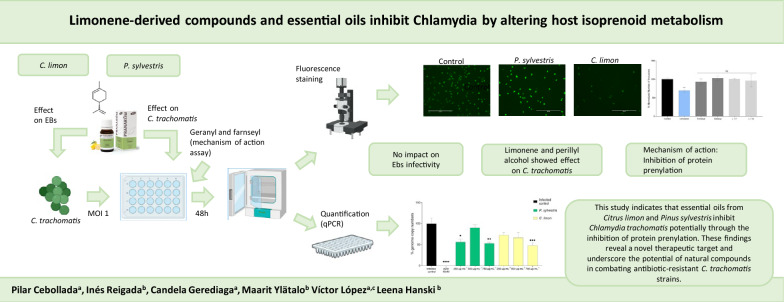

**Supplementary Information:**

The online version contains supplementary material available at 10.1007/s13659-026-00611-5.

## Introduction

*Chlamydia trachomatis* is an obligate intracellular pathogen, most notably for causing sexually transmitted infections (STIs) and ocular infections. This bacterium exhibits multiple serovars, among which A to C are responsible for trachoma, a form of infectious blindness, while D to K represent the most common bacterial STIs worldwide and L1 to L3 usually lead to lymphatic system infections. *C. trachomatis* infections may cause cervicitis, fibrotic endometritis, and pelvic inflammatory disease in women. Vertical transmission during delivery can result in neonatal conjunctivitis [1, 2]. Most infected women and half of infected men are asymptomatic, which complicates the management as the non-treated infection may progress, leading to a chronification with severe consequences such as pelvic inflammatory disease, ectopic pregnancy, and obstructive infertility [3]. People unaware of their infection may also risk their sexual partners' health, therefore contributing to the pathogen dissemination. In fact, in a context where the incidence of STIs is increasing, it is worth to highlight that *C. trachomatis* infections represent the most prevalent bacterial STI globally, with 131 million new cases annually [4].

*C. trachomatis* exhibits a distinctive lifecycle, in which the bacteria can exist primarily in two different forms: the infectious elementary body (EB) and the reproductive reticulate body (RB). EBs, the extracellular forms, are responsible for the infection of the host cells. EBs internalize into the host eukaryotic cell inside endocytic vacuoles which fuse and mature to form an inclusion where the EBs transform into RBs, the replicative form of *C. trachomatis* [5]. RBs replicate by binary fission utilizing the host cell nutrients, filling the cytoplasm and dislocating the nucleus. Besides, the bacteria exploit host vesicular trafficking by recruiting and modulating Rab GTPases, to facilitate their intracellular survival and replication [6]. RBs convert back into EBs, which are then released into the extracellular space either by host cell lysis or by extrusion of the intact inclusion, allowing them to infect new host cells [7].

Under stress conditions such as antibiotic or interferon-γ exposure, nutrient deprivation, or coinfection, *C. trachomatis* can enter a persistent state marked by enlarged, non-infectious aberrant RBs, resulting in more complex management of the infection [8, 9]. Apart from the ability to enter persistent state, *C. trachomatis* also presents a challenge for treatment due to antibiotic resistance, a problem common among many other bacteria. *C. trachomatis* infections are usually treated with inhibitors of the protein synthesis, namely tetracyclines (doxycycline) and macrolides (azithromycin). Despite its ability to induce chlamydial persistence, in some cases amoxicillin is also considered as a therapeutic option [10]. Reported failure rates range from 5 to 23%, often linked to resistance driven by prolonged exposure to subinhibitory drug concentrations [11]. Besides the two lipid membranes of the gram-negative bacterium itself, the host-derived inclusion membrane and host cell plasma membrane constitute additional permeability barriers which limit the local antibiotic concentrations reaching the replicating chlamydial RBs. These challenges underscore the need for alternative antimicrobials and novel treatment strategies.

Essential oils (EOs) are plant-derived volatile products consisting of a mixture of terpenes, sesquiterpenes and aromatic compounds, typically obtained through steam or hydro distillation or in the case of citrics, by mechanical pressure. The antimicrobial properties of EOs and individual EO-derived terpenes have been extensively studied, indicating that most of these lipophilic compounds exert direct bactericidal effects through disruption of microbial membranes and subsequent loss of membrane polarization. To date, data on the impact of EOs or EO-derived terpenes on intracellular bacteria have remained very scarce, presumably due to often limited selectivity in targeting prokaryotic versus eukaryotic membranes and hence cytotoxicity on host cells. In the case of *Chlamydia*, chances for selective chlamydiocidal activity are further compromised due to presence of host-derived lipids in both bacterial plasma membrane and outer membrane. Two previous studies have addressed the effects of EOs and EO-derived terpens on EB infectivity [12–14] and one study has suggested an inhibitory effect of a *Mentha* EO on RB replication stage [14].

The current study was established with the hypothesis that identifying non-toxic EOs capable of suppressing *C. trachomatis* growth during the intracellular stages of its life cycle can guide the identification of antichlamydial terpenes with mechanisms of action distinct from direct disruption of chlamydial membranes. Following this reasoning, we identified limonene as a previously unknown *C. trachomatis* growth inhibitor based on its dominant proportion in the composition of two commercially available antichlamydial EOs, *Citrus limon* and *Pinus sylvestris.* Limonene was found to suppress *C. trachomatis* intracellular growth and progeny production but not affect EB infectivity. Drawing on the known ability of limonene and its metabolite perillyl alcohol to suppress protein prenylation in eukaryotic cells, we hypothesized a role of altered host protein prenylation in the observed antichlamydial effect and conducted supplementation experiments with the prenylation substrates farnesyl and geranylgeranyl to experimentally test this concept.

## Materials and methods

### Chemicals

The EOs selected for this work were obtained from Pranarom (Ghislenghien, Belgium). The main characteristics of the EOs are listed in Table 1.

**Table 1:** Summary of the key characteristics of the EOs studied in this work, including their name, batch, plant part used, country of origin, and primary compounds

Scientific name	Name	Batch	Country	Part of the plant
*Citrus limon*	Lemon	OF005051	Argentina	Zest
*Pinus sylvestris; L*	Pine	OF42911	Bulgaria	Needles

**Fig. 1:**
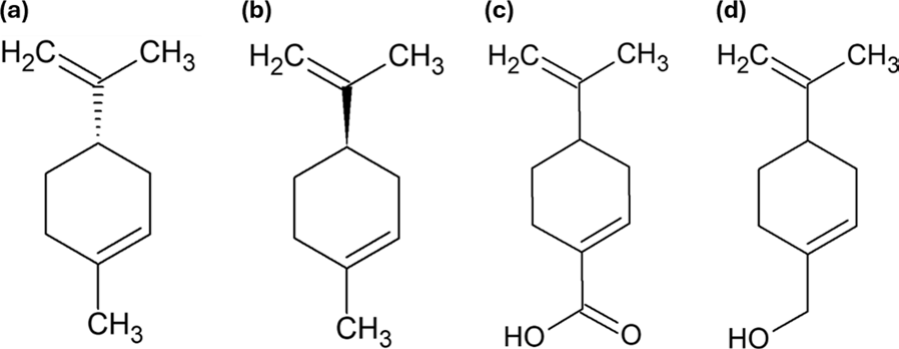
Chemical structures of **a** (S)-(−)-limonene, **b** (R)-(+)-limonene, **c** perillic acid, and **d** perillyl alcohol. Structures were generated using ChemSketch 2.0

S-(−)-limonene and R-(+)-limonene (Sigma-Aldrich, St. Louis, MO, US) (Fig. 1) were used to compare its activity with the EOs. For cell culture, Dulbecco’s Modified Eagle’s Medium (DMEM; Cytiva, Marlborough, Massachusetts, United States), gentamicin (Sigma-Aldrich, St. Louis, MO, US), and heat-inactivated fetal bovine serum (FBS; Invitrogen, Thermo Scientific, Waltham, MA, US) were used. Azithromycin (AZM; Cayman Chemical, Ann Arbor, MI, US) served as a positive control in antichlamydial assays. For bacterial genome quantification, the GeneJET Genomic DNA Purification Kit (Thermo Fisher Scientific, Massachusetts, USA), primers, and Master Mix (Applied Biosystems, Thermo Scientific, Waltham, MA, US) were used. For inclusion staining, Chlamydia LPS Monoclonal Antibody (B410F), Goat anti-Mouse IgG (H + L) Highly Cross-Adsorbed Secondary Antibody (Invitrogen, Thermo Scientific, Waltham, MA, US), Alexa Fluor™ Plus 488 (Invitrogen, Thermo Scientific, Waltham, MA, US), Evans Blue, Propidium monoazide (PMA) and cycloheximide (Sigma-Aldrich, St. Louis, MO, USA) were used. The antichlamydial activity of perillyl alcohol (Sigma-Aldrich, St. Louis, MO, US) and perillic acid (Santa Cruz Biotechnology), metabolites of limonene, was also evaluated. The chemical structure of limonene and its metabolites herein tested is presented in Fig. 1. For mechanism of action studies, farnesyl pyrophosphate ammonium salt and geranylgeranyl pyrophosphate ammonium salt (Merck Life Sciences) were obtained.

### Chemical characterization

The chemical composition of both EOs was determined using gas chromatography coupled with mass spectrometry (GC–MS). Analyses were performed on an Agilent Technologies GC 6890 system interfaced with a 5973-mass selective detector and fitted with an HP-5MS-IU capillary column (30 m × 0.25 mm, 0.25 µm film thickness; Agilent Technologies). Samples were introduced in split mode at a ratio of 100:1, with an injection volume of 0.2 µL.

The oven temperature program began at 60 °C and was ramped to 240 °C at a rate of 3 °C per minute. Helium served as the carrier gas at a constant flow rate of 1.0 mL/min. Mass spectrometric detection was conducted in electron impact (EI) mode at 70 eV, scanning over an m/z range of 41–250. Compound identification was achieved by comparing both retention times and mass spectra with reference data from the NIST library and previously published literature [15].

Additionally, linear retention indices (LRIs) were calculated using a homologous series of n-alkanes (C8–C30) run under identical conditions. These experimental indices were cross-referenced with literature values to support the provisional identification of analytes. Where possible, compound assignments were further corroborated by matching both fragmentation patterns and LRIs with those reported in the Wiley275 library and by Adams (2009).

### Bacterial strains

The chlamydial species utilized was an isolate of *C. trachomatis*, Serovar K (ATCC VR-887). It was propagated in HeLa-229 (Homo sapiens, human epithelial cells; HeLa-229 ATCC (CCL-2.1); LOT: 59501359) cells and stored in 0.2 M sucrose, 0.02 M sodium phosphate (pH 7.4) and 5 mM glutamic acid (SPG-buffer) at − 80 °C until used.

### Mammalian cells maintenance and cytotoxicity assessment

HeLa-229 cells were cultured in DMEM, supplemented with 20 µg mL^−1^ gentamicin and 10% FBS. The cells were maintained at 37 °C with 5% CO_2_ in a humidified incubator (Heracell^™^ 240i, Thermo Scientific, Waltham, MA, US). The viability of HeLa cells upon exposure to EOs, limonene, perllyl alcohol and perillic acid was determined by seeding HeLa-229 cells into 96-well plates (6 × 10^4^ cells/well) and incubating them for 24 h before treatment with various substance concentrations. After 48 h, cells were washed with PBS, stained with 20 µM resazurin, and incubated for 2 h. Fluorescence (λ_ex_ = 560 nm, λ_em_ = 590 nm) was recorded using a Varioskan LUX microplate reader (Thermo Scientific, Waltham, MA, US).

### Determination of activity against *C. trachomatis*

#### Infection procedure

HeLa-229 cells were plated in 24-well plates (4 × 10^5^ cells/well) on glass coverslips. After a 24 h incubation, the cells were infected with *C. trachomatis*, Serovar K (ATCC VR-887) at a multiplicity of infection (MOI) of one in infection media (DMEM + Cycloheximide 1 µg mL^−1^) and the plate was centrifugated for 1 h at 550 g and further incubated for another hour at 37 °C.

#### Activity against *C. trachomatis*

To study the effect of the EOs, limonene, metabolites and AZM 40 nM (positive control) on *C. trachomatis*, the infection procedure described above was followed. After the 1 h incubation at 37 °C, the inoculum was replaced with fresh infection media with different concentrations of EO. The plate was then further incubated for 48 h.

#### Infectious progeny assay

To assess the impact on chlamydial infectious progeny production, the procedure described in 2.5.2 was followed. Following incubation, the infected cells were detached from the wells and frozen at −80 °C to enable the lysis. The cell lysate was then used to infect fresh HeLa-229 cells in a 24-well plate as in 2.5.1.

#### Direct impact on EBs

*C. trachomatis* (4 × 10^5^ Inclusion Forming Units) was pretreated with different concentrations of EOs or limonene in infection media and incubated for 1 h at 4 °C. After this pretreatment, the EOs and AZM were removed in a 1 h centrifugation (4 °C and 21 000 g). Subsequently, the EBs were resuspended in fresh medium and inoculated into the HeLa-229 cells, as described in 2.5.1.

#### DNA extraction and quantitative PCR measurement

Following incubation, the cells were scraped and centrifuged at 2000 × g for 5 min, resuspended and stored at − 80 °C. Genomic DNA was extracted using the GeneJET Genomic DNA Purification Kit. Quantification was performed using a StepOnePlus^™^ Real-Time PCR System with StepOne^™^ Software v2.3 (Thermo Fisher Scientific, Waltham, MA, USA). Each qPCR reaction contained 20 ng of template DNA, Fast SYBR^™^ Green Master Mix, and 0.4 mM of each primer (Forward: GGCGTATTTGGGCATCCGAGTAACG; Reverse: TCAAATCCAGCGGGTATTAACCGCT), in 96-well MicroAmp optical plates (Thermo Fisher Scientific). Thermal cycling was set to 95 °C for 20 s, followed by 40 cycles of 95 °C for 3 s and 60 °C for 30 s. A melting curve analysis followed, consisting of 95 °C for 15 s, 60 °C for 1 min, and 95 °C for 15 s. Each run included non-template and negative controls. Standard curves for genome equivalent (GE) quantification were generated by serial dilution of purified *C. trachomatis* DNA with a known titer.

#### Inclusion staining

At 48 h post-infection, cells grown on coverslips were washed with PBS, fixed in methanol for 10 min, and blocked with 0.5% BSA for 15 min. They were then incubated with 75 µL of Chlamydia LPS Monoclonal Antibody (B410F) diluted 1:100 for 1 h in a humid chamber. After washing, Goat anti-Mouse IgG (H + L), Alexa Fluor^™^ Plus 488 diluted 1:2000, was applied. Following a second wash, cells were stained with 100 µL of Evans Blue for 20 min, rinsed three times with Milli-Q water, and then analyzed under a microscope.

### Mechanism of action studies

#### EBs viability PCR

To complement the EB infection-based assays and evaluate whether the EOs and limonene affected its membrane, the viability-PCR (V-PCR) approach described by Janssen et al. was adapted [16]. Briefly, EBs suspensions were incubated for 1 h at 4 °C in the presence EOs (750 µg mL^−1^), or R(+)-limonene (525 µg mL^−1^). Untreated EBs were included as viability controls. Following incubation, samples were centrifuged at 21,000 × g for 1 h at 4 °C and resuspended in 500 µL SPG buffer. Two additional EB aliquots were heat-killed (95 °C, 10 min) to serve as non-viable controls. PMA was added to all preparations to a final concentration of 50 µM, and samples were incubated for 10 min in the dark at 4 °C, followed by light activation for 15 min at 465 nm. After a final centrifugation step (21,000 × g, 1 h, 4 °C), DNA was extracted and subjected to qPCR as described in Sect. 2.5.5. A marked reduction in amplification signal was interpreted as loss of EB viability due to compromised membrane integrity. An infected control non-treated and a heat-treated control were prepared to confirm the assay’s reliability.

#### Inhibition of protein prenylation

To determine whether the antichlamydial effect of limonene and perillyl alcohol is mediated through the inhibition of protein prenylation, cell cultures were supplemented with farnesyl pyrophosphate and geranylgeranyl pyrophosphate (substrates of prenyltransferase enzymes) to assess whether the additional substrate could counteract limonene’s antichlamydial activity. To do so, infection with *C. trachomatis* was performed as explained in Sect. 2.5.1. After that, cells were treated with 10 µM of farnesyl pyrophosphate or geranylgeranyl pyrophosphate alone or in combination with R-(+) limonene (525 µg mL^−1^) or perillyl alcohol (100 µg mL^−1^).

### Statistical analysis

Statistical analyses were performed using GraphPad Prism 10, with outliers identified via Grubbs’ test. One-way ANOVA followed by Dunnett’s or Bonferroni’s post hoc tests was used to assess differences between sample concentrations and the control (*p*-values reported). For bacterial inclusion quantification, images were processed using a custom Python script employing libraries such as skimage, numpy, matplotlib, and pandas. The script converted images to grayscale, applied Gaussian filtering, thresholding, and morphological operations, and labelled regions to detect and count fluorescent signals, compiling the results into a DataFrame for statistical analysis performed as previously described. GraphPad Prism 10 was also used to create the Figures.

## Results

### The impact of *C. limon* and *P. sylvestris *EOs on *C. trachomatis* growth and progeny production

**Fig. 2:**
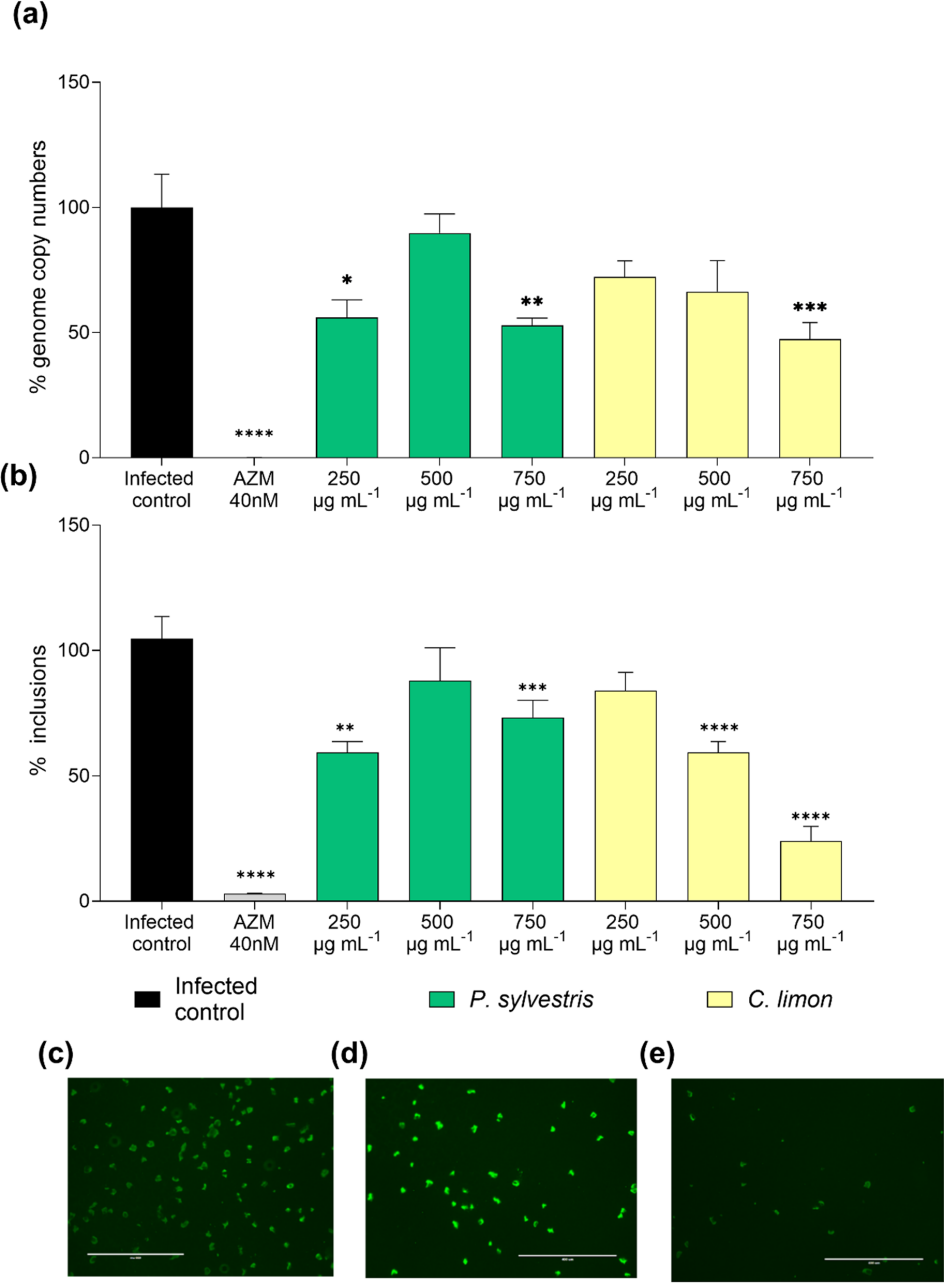
Impact of EOs on *C. trachomatis* growth. **a** Percentage of genome copy numbers and **b** percentage of inclusions of *C. trachomatis* remaining after treatment with *C. limon* and *P. sylvestris* EOs. Data were normalized to the untreated control. Different concentrations of EOs ranging from 250 to 750 µg mL^−1^ were studied. Mean and SEM values are displayed, n = 4. Representative images for each condition are presented in panels **c**, **d**, and **e**, corresponding to the untreated control, treatment with *P. sylvestris* EO (750 µg mL^−1^) and treatment with *C. limon* EO (750 µg mL^−1^) respectively. Significant differences were determined by ANOVA (statistical significance: *p < 0.05; **p < 0.01; ***p < 0.001; ****p < 0.0001)

In our search for antichlamydial EOs showing no cytotoxicity on the HeLa-229 epithelial cell line serving as the *C. trachomatis* infection host, *C. limon* and *P. sylvestris* EOs stood out (Supplementary Information). The impact of EOs on *C. trachomatis* growth in HeLa-229 cells was studied by administering the EOs after *C. trachomatis* EB internalization (starting the treatment at 2 h post-infection). At the end of a 48-h infection, both bacterial genome copy numbers and bacterial inclusion counts were quantified. *C. limon* reduced the genome copy numbers in a dose-dependent manner, showing a 27.7% and 33.6% reduction at 250 and 500 µg mL^−1^ respectively (Fig. 2a). A statistically significant reduction in *C. trachomatis* genome copy numbers was reached at the highest concentration (52.6% reduction; *p* < 0.001). *C. limon* EO also reduced the number of chlamydial inclusions (Fig. 2b) at 500 (40.6% reduction;* p* < 0.001) and 750 µg mL^−1^ (76.1% reduction;* p* < 0.001). Figure 2 also shows that *P. sylvestris* also displayed activity at some of the concentrations but did not show a concentration-dependent activity within the tested conditions.

**Fig. 3:**
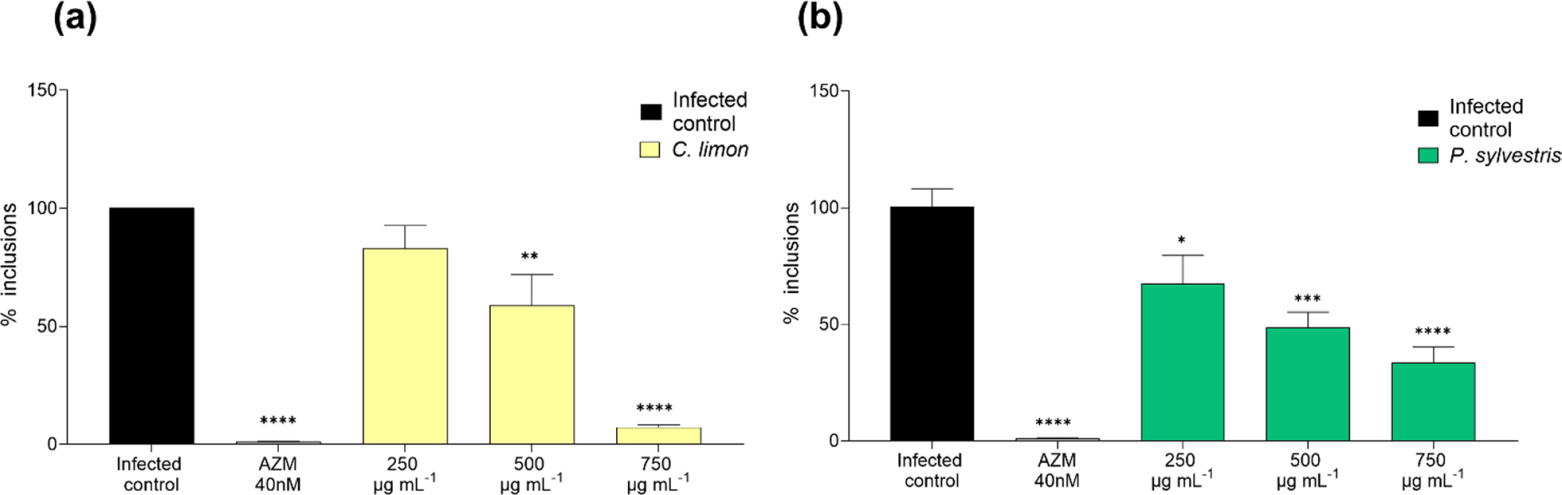
Impact of *C. limon* and *P. sylvestris* EOs on *C. trachomatis* infectious progeny production. The figure shows the percentage of bacterial inclusion of *C. trachomatis* in HeLa-229 cells infected with the infectious progeny obtained from a first infection in another set of HeLa-229 cells treated with *C. limon* (**a**) and *P. sylvestris* (**b**) at different concentrations (250–750 µg mL^−1^), as well as azithromycin (AZM) used as a positive control and untreated controls. Data were normalized to the untreated control. Mean and SEM values are displayed, n = 4. Significant differences were determined by the ANOVA test (statistical significance, when compared to the untreated control: *p < 0.05; **p < 0.01. ***p < 0.001. ****p < 0.0001)

The effects of both EOs on preventing the generation of infectious *Chlamydia* progeny was determined by infecting fresh HeLa-229 cells with lysates collected from EO-treated infections. *C. limon* EO reduced the number of inclusions in the infectious progeny assay a 16.9%, 41.0% and 92.7% at concentrations of 250 µg mL^−1^, 500 µg mL^−1^ (*p* < 0.01) and 750 µg mL^−1^ (*p* < 0.0001) respectively (Fig. 3a). *P. sylvestris* proved to be active (*p* < 0.05, *p* < 0.001 and *p* < 0.0001) at the same concentrations reducing the number of inclusions by 32.5%, 51.3% and 66.4% respectively (Fig. 3b).

### Chemical composition of the EOs

The chemical composition of both EOs was determined using gas chromatography coupled with mass spectrometry GC–MS. 29 compounds were identified in *C. limon* EO, with limonene representing the 64.13% of the EO (Table 2). Other compounds found in the EO were gamma-terpinene (11.022%) and beta-Pinene (10.767%). For *P. sylvestris*, a total of 46 compounds were identified (Table 3). The three major constituents were alpha-pinene (32.06%), beta -pinene (19.63%), and limonene + beta -phellandrene (11.52%). Chemical structures of the main compounds found in both EO are presented in the Supplementary Information.

**Table 2:** Chemical characterization of *C. limon* EO

Compound	IA^a^	CAS number	%
Tricyclene	922	508-32-7	0.010
Alfa-Thujene	926	28167-05-2	0.593
Alfa-Pinene	933	80-56-8	2.464
Camphene	947	79–92-5	0.082
Sabinene	972	3387-41-5	2.555
**Beta-Pinene**	**976**	**127-91-3**	**10.767**
Myrcene	992	123-35-3	1.370
Pseudolimonene	1005	499-97-8	0.098
Alfa-Terpinene	1017	99-86-5	0.320
p-Cymene	1025	99-87-6	0.228
**Limonene**	**1028**	**138-86-3**	**64.125**
(E)-beta-Ocimene	1047	3779-61-1	0.085
**Gamma-Terpinene**	**1058**	**99-85-4**	**11.022**
Terpinolene	1089	586-62-9	0.450
Linalool	1103	78-70-6	0.166
Camphor	1143	76-22-2	0.044
Citronellal	1155	106-23-0	0.058
Borneol	1163	464-45-9	0.040
Terpinen-4-ol	1178	562-74-3	0.058
Alfa-Terpineol	1179	98-55-5	0.350
Nerol	1231	106-25-2	0.067
Neral	1241	106-26-3	0.722
Geranial	1271	141-27-5	1.431
Neryl acetate	1366	141-12-8	0.410
Beta-Caryophyllene	1417	87-44-5	0.520
Trans-alfa-Bergamotene	1435	13474-59-4	0.723
Alfa-Humulene	1452	6753-98-6	0.064
Bicyclogermacrene	1495	67650-90-2	0.138
Beta-Bisabolene	1508	495-61-4	0.667

^a^Adjusted Retention Index; Compounds shown in bold represent the major constituents.

**Table 3:** Chemical characterization of *P. sylvestris* EO

Compound	IA^a^	CAS number	%
Tricyclene	922	508-32-7	1.194
Alfa-Thujene	926	28167-05-2	0.125
**Alfa-Pinene**	**933**	**80-56-8**	**32.056**
Camphene	947	79–92-5	4.757
Sabinene	973	3387-41-5	0.116
**Beta-Pinene**	**976**	**127-91-3**	**19.627**
Myrcene	992	123-35-3	4.035
Alfa-Phellandrene	1005	99-83-2	0.092
Delta-3 Carene	1011	13466-78-9	1.239
Alfa-Terpinene	1017	99-86-5	0.082
p-Cymene	1024	99-87-6	0.122
**Limonene + beta-Phellandrene**	**1028**	**138-86-3**	**11.518**
Cis-beta-Ocimene	1034	3338-55-4	0.079
Trans-beta-Ocimene	1047	3779-61-1	0.892
Gamma-Terpinene	1058	99-85-4	0.138
Terpinolene	1089	586-62-9	0.467
Fenchol	1113	1632-73-1	0.106
Trans-Pinocarveol	1137	547-61-5	0.206
Borneol	1163	464-45-9	0.101
Terpinen-4-ol	1179	562-74-3	0.100
Alfa-Terpineol	1189	98-55-5	0.519
Myrtenol	1195	515-00-4	0.208
Verbenone	1208	80-57-9	0.048
Thymol methyl ether	1235	1076-56-8	0.083
Bornyl acetate	1285	76-49-3	2.284
Alfa-Cubebene	1349	17699-14-8	0.479
Alfa-Terpenyl acetate	1350	80-26-2	0.001
Alfa-Copaene	1374	3856-25-5	0.305
Beta-Bourbonene	1384	5208-59-3	0.114
beta-Cubebene	1390	13744-15-5	0.065
beta-Elemene	1392	515-13-9	0.383
(E)-beta-Caryophyllene	1417	87-44-5	3.071
Aromadendrene	1437	489-39-4	0.266
Alfa-Humulene	1452	6753-98-6	0.575
Bicyclosesquiphellandrene	1461	54324-03-7	0.195
Alfa-Amorphene	1476	23515-88-0	0.615
Germacrene-D	1480	23986-74-5	1.242
Beta- Selinene	1485	17066-67-0	0.566
Bicyclogermacrene	1495	67650-90-2	0.498
Alfa-Muurolene	1499	31983-22-9	1.165
Gamma-Cadinene	1513	39029-41-9	1.859
Delta-Cadinene	1524	483-76-1	4.098
Spathulenol	1576	77171-55-2	0.444
Caryophyllene oxide	1584	1139-30-6	0.298
Alfa-Cadinol	1644	481-34-5	0.523
T-Cadinol	1654	5937-11-1	0.420

^a^Adjusted Retention Index; Compounds shown in bold represent the major constituents.

### Impact of limonene on *C. trachomatis* growth

**Fig. 4:**
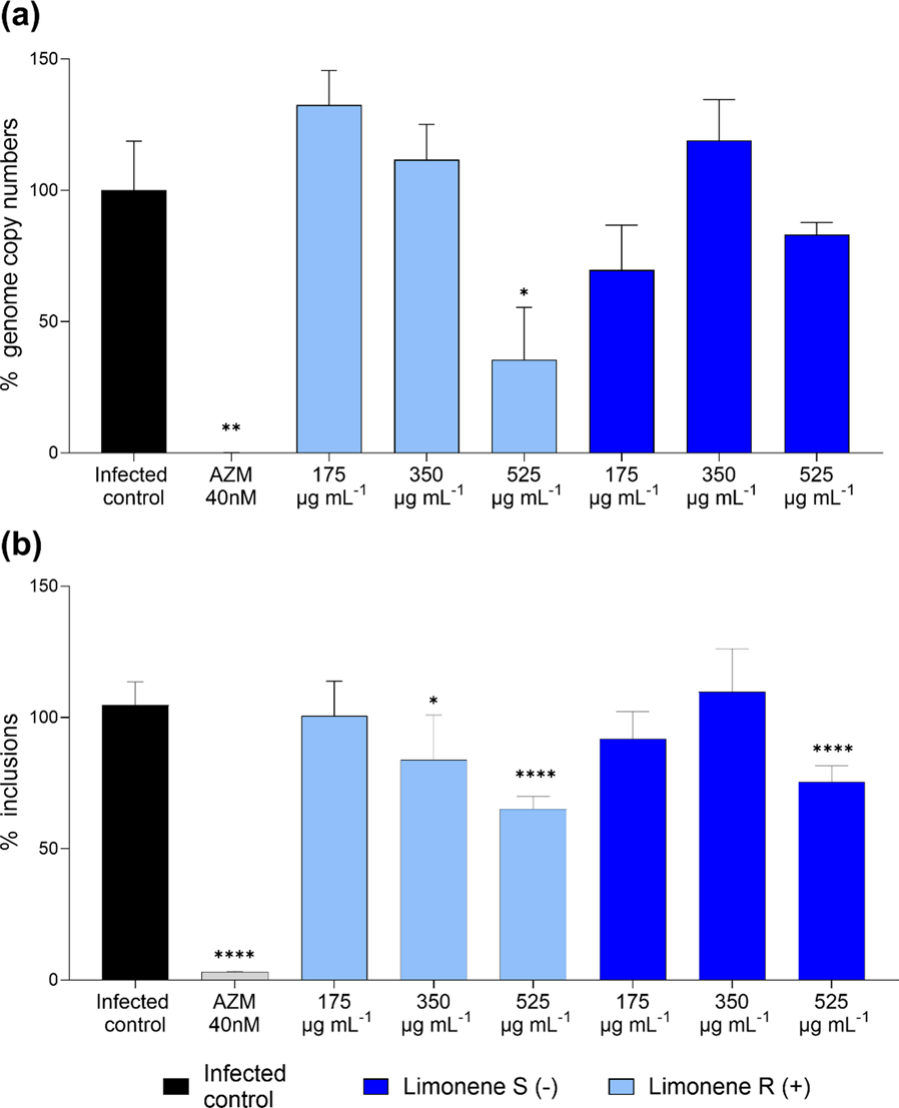
Impact of limonene on *C. trachomatis* growth. **a** Remaining genome copy numbers and **b** remaining inclusion counts of *C. trachomatis* after treatment with the R and S enantiomers of limonene. Data were normalized to the untreated control. Different concentrations of limonene ranging from 175–525 µg mL^−1^ were studied. In the graphs mean and SEM values are displayed, n = 4. Significant differences were determined by ANOVA (statistical significance: *p < 0.05; **p < 0.01; ***p < 0.001; ****p < 0.0001)

Given its remarkable proportion in the composition of both studied EOs, the impact of commercially obtained pure limonene was studied under the same conditions against *C. trachomatis*. Limonene concentrations were adjusted to correspond to its quantities present in *C. limon* EO at concentrations exhibiting antichlamydial activity. The impact of both enantiomers on *C. trachomatis* growth was studied as regard to genome copy numbers (Fig. 4a) and to bacterial inclusion counts (Fig. 4b). In both cases, R-Limonene showed a higher reduction than the one displayed by S-Limonene. In fact, the highest R-limonene concentration 525 µg mL^−1^ significantly reduced the genome copy numbers of *C. trachomatis* (64.5% reduction; *p* < 0.05) (Fig. 4a). Both S-Limonene and R-Limonene dose-dependently reduced the number of chlamydial inclusions (Fig. 4b). The inhibitory effect reached statistical significance for S-limonene at the highest concentration 525 µg mL^−1^ (24.64% reduction; *p* < 0.0001), while R-limonene showed reduction for both 350 µg mL^−1^ (16.2% reduction; *p* < 0.05) and 525 µg mL^−1^ (34.9% reduction, *p* < 0.0001).

**Fig. 5:**
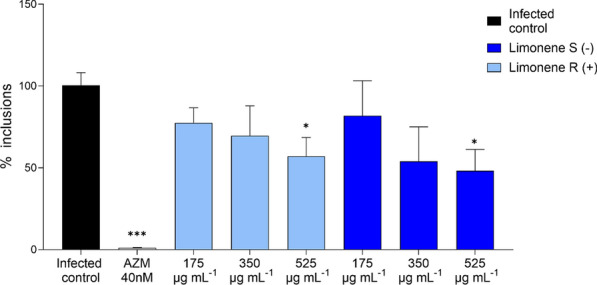
Impact of limonene on the infectious progeny. The figure shows the percentage of remaining bacterial inclusion counts of *C. trachomatis* in HeLa-229 cells infected with the infectious progeny obtained from a first infection in another set of HeLa-229 cells treated with limonene (175–525 µg mL^−1^), azithromycin (AZM) and untreated controls. Data were normalized to the untreated control, with mean values and SEM being displayed (n = 4). Significant differences were determined by ANOVA (statistical significance: *p < 0.05; **p < 0.01. ***p < 0.001. ****p < 0.0001)

The impact of limonene on the *C. trachomatis* infectious progeny production was also studied for both S-limonene and R-limonene (Fig. 5). For both enantiomers, the effect was dose-dependent and reached statistical significance (*p* < 0.05) at the highest concentration (525 µg mL^−1^), reducing the number of chlamydial inclusions by 47.9% for R-limonene and 51.8% for S-limonene.

### Impact of limonene and the limonene-containing EOs on *C. trachomatis* EBs

**Fig. 6:**
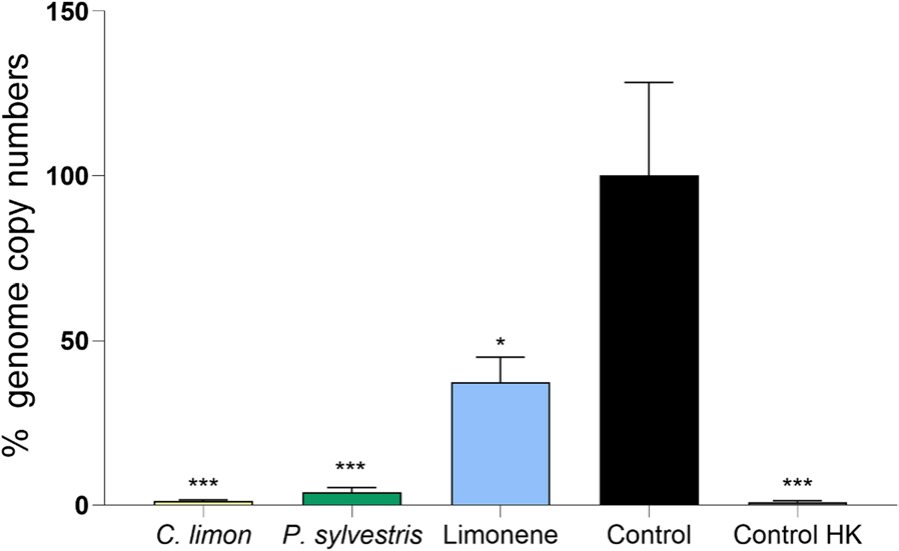
Viability-PCR analysis of *C. trachomatis* EBs after treatment with essential oils and limonene. Percentage of *C. trachomatis* genome copy numbers obtained by Viability-PCR after EB treatment with *C. limon* and *P. sylvestris* EOs (750 µg mL⁻^1^) or R (+)-limonene (525 µg mL⁻^1^). A heat killed (HK) EBs were used as a positive control. Data were normalized to the untreated control, with mean values and SEM being displayed (n = 4). Significant differences were determined by ANOVA (statistical significance: *p < 0.05; **p < 0.01. ***p < 0.001. ****p < 0.0001)

To determine whether limonene affected the *C. trachomatis* EB membrane, viability-PCR was performed. In this assay, EB suspensions are exposed to the studied substances for 1 h and subsequently exposed to propidium monoazide (PMA). EBs with compromised membrane integrity allow PMA to enter the cell and intercalate with the DNA, thereby preventing DNA amplification in the subsequent PCR reaction. As shown in Fig. 6, when V-PCR is performed, significant differences were found between the infectious control and the samples. Both EOs at 750 µg mL⁻^1^ significantly reduced the genome copy numbers (*p* < 0.001). Also, the pure limonene affected EB membranes (*p* < 0.05), although the effect was less pronounced than the one exhibited by the EOs.

**Fig. 7:**
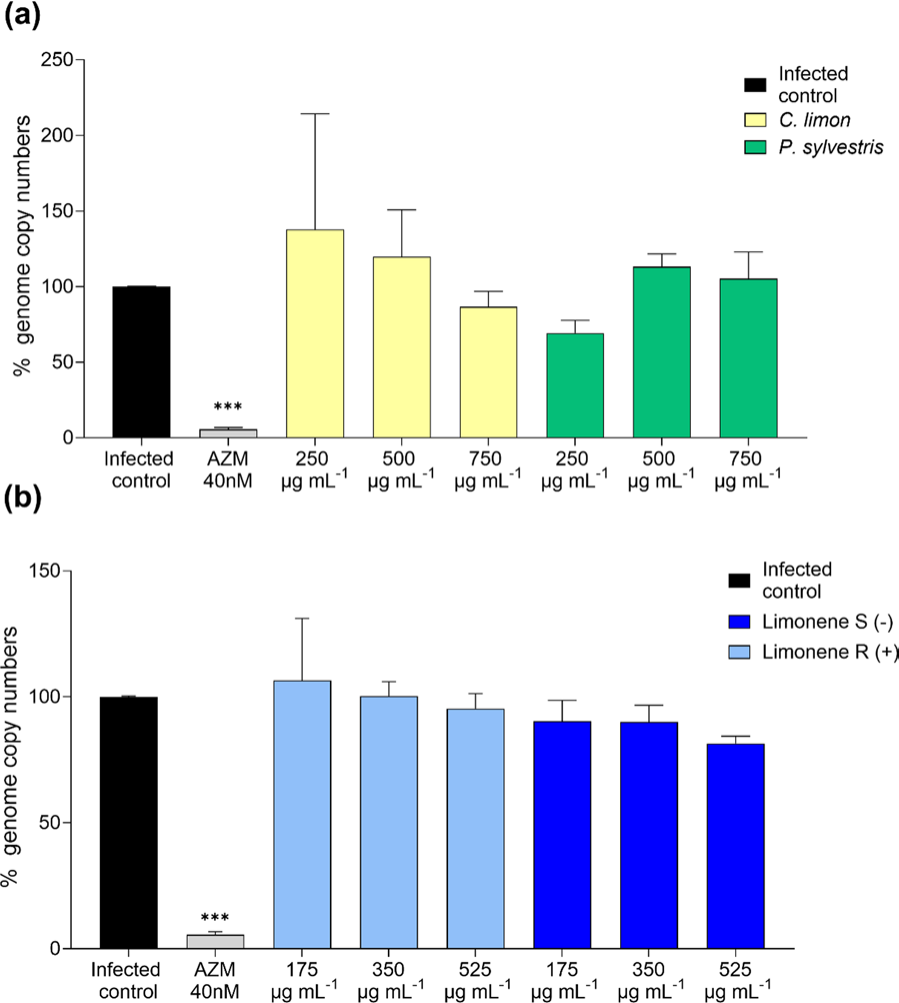
Impact of the *C. limon* and *P. sylvestris* EOs on C. trachomatis EB infectivity. Genome copy numbers of *C. trachomatis* were obtained from HeLa-229 cells infected with EBs pre-treated with EOs (**a**) or limonene (**b**) at different concentrations. Azithromycin (AZM) was used as a positive control. Data were normalized to untreated control. Mean and SEM values are displayed, n = 4. Statistical analysis with ANOVA (statistical significance: *p < 0.05; **p < 0.01. ***p < 0.001. ****p < 0.0001)

Since the viability-PCR data indicated that limonene and limonene-containing EOs may affect *C. trachomatis* EB membrane permeability (Fig. 6), we wanted to determine whether this effect translates to altered EB infectivity. To this end, EB suspensions were exposed to limonene or the EOs for 1 h before inoculating them to HeLa-229 monolayers. In Fig. 7, the impact of both EOs (Fig. 7a) and limonene enantiomers (Fig. 7b) on the *C. trachomatis* EB infectivity is depicted. The results showed that neither the pure limonene enantiomers nor the limonene-containing EOs have significant effect decreasing the EB infectivity. Only the positive control azithromycin showed significant differences (*p* < 0.0001)*.*

### Involvement of host protein prenylation in the antichlamydial activity

**Fig. 8:**
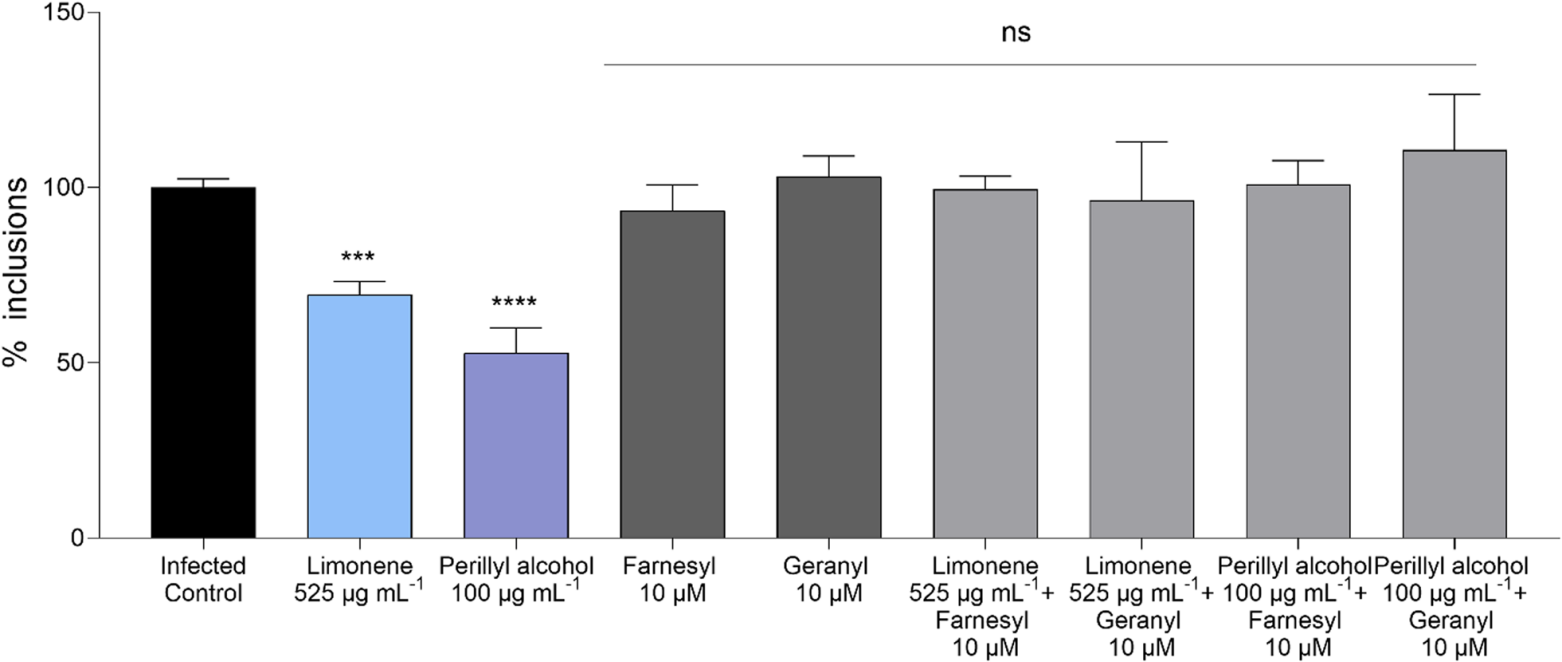
Impact of Farnesyl pyrophosphate and Geranylgeranyl pyrophosphate supplementation on the antichlamydial activity of limonene and perillyl alcohol. The figure shows the percentage of remaining inclusions of *C. trachomatis* after the treatment with R-limonene (525 µg mL^−1^), perillyl alcohol (100 µg mL^−1^), farnesyl (10 µM), geranylgeranyl (10 µM), limonene (525 µg mL^−1^) + farnesyl (10 µM), limonene (525 µg mL^−1^) + geranylgeranyl (10 µM), perillyl alcohol (100 µg mL^−1^) + farnesyl (10 µM) and perillyl alcohol (100 µg mL^−1^) + geranylgeranyl (10 µM). Data were normalized to untreated control. Mean and SEM values are displayed (n ≥ 4). Significant differences between groups were determined using ANOVA followed by Dunnett’s post hoc correction for multiple comparisons (statistical significance, when compared to the infected control: statistical significance: *p < 0.05; **p < 0.01. ***p < 0.001. ****p < 0.0001, ns: non-significant). No significant differences between the infected control and the perillic acid were found (not represented)

As the above-described data indicated that direct targeting of chlamydial membranes does not contribute to the observed antichlamydial effect, we tested an alternative hypothesis on the underlying mechanism, namely the involvement of host cell altered protein prenylation. To this end, two interconnected approaches were taken. First, the impact of two limonene metabolites, perillyl alcohol and perillic acid were evaluated for their ability to reduce the number of chlamydial inclusions. While no significant differences between the infected control and the perillic acid were found (data not shown), perillyl alcohol (100 µg mL^−1^) reduced the inclusion counts by 66.2% (*p* < 0.0001; Fig. 8).

Second, the impact of supplementing the cultures with external prenyltransferase substrates was used to assess the involvement of possible competitive inhibition of this cellular process. The chlamydial growth inhibition experiment with both limonene and perillyl alcohol were repeated under the same conditions as in previous experiments, adding farnesyl pyrophosphate and geranylgeranyl pyrophosphate to the cultures. The results shown in Fig. 8 demonstrate that both limonene and perillyl alcohol consistently reduce the number of *C. trachomatis* inclusions. The addition of farnesyl pyrophosphate or geranylgeranyl pyrophosphate alone did not affect inclusion counts (Fig. 8). However, when perillyl alcohol and limonene-treated infections were supplemented with either of the lipids, the inhibitory effect of both limonene and its metabolite were almost entirely lost, showing no significant differences with the infected control.

## Discussion

Although terpenes and EOs have been widely studied for their bioactive properties, their activity against intracellular bacteria remains mostly unexplored. While *C. trachomatis* is an obligate intracellular pathogen, several non-obligate bacteria can also adopt an intracellular lifestyle as a virulence strategy, gaining access to nutrients, evading immune responses, and establishing reservoirs that contribute to persistence and reinfection [17]. This highlights the relevance of exploring therapeutic approaches targeting intracellular bacterial forms. The results of the current study indicate that limonene, a monoterpene found in various EOs, inhibits *C. trachomatis* intracellular growth and progeny production but does not significantly reduce *C. trachomatis* EB infectivity in vitro. Furthermore, perillyl alcohol, the main metabolite of limonene, was found to be more potent *C. trachomatis* growth inhibitor than its parent compound, and the antichlamydial effect of both limonene and perillyl alcohol was found to be abolished by supplementing the infections with protein prenyltransferase substrates.

The identification of limonene as an antichlamydial compound was guided by the observation that two limonene-rich EOs, namely lemon and pine, showed antichlamydial activity when administered to epithelial cells after *C. trachomatis* EB internalization. Lemon essential oil, widely used in cosmetics, hygiene products, foods and aromatherapy, has been extensively studied for its bioactive properties and found to exhibit diverse properties including antimicrobial activity [18, 19]. Pine trees, widely found in Europe, Asia, North America and North Africa, yield EOs which have been reported to carry various bioactivities, including antiviral and antibacterial effects [20]. While we found *P. sylvestris* EO to reproducibly reduce *C. trachomatis* growth, its impact on both bacterial genome copy numbers and chlamydial inclusion counts did not show clear concentration-dependence and was lower than that displayed by *C. limon* EO.

For both studied EOs, the efficacy seemed to be lower for the genome copy numbers compared to the reduction of inclusion counts under the same conditions. This may reflect the fact that qPCR is able to detect DNA also from non-viable *C. trachomatis*, indicating that the presence of genetic material might not necessarily reflect the intracellular bacterial viability [16].

A recent study reported that *P. sylvestris* and *C. limon* EOs, as well as their major constituent limonene are active against drug-resistant *Neisseria gonorrhoeae* [21]. *N. gonorrhoeae* a is a sexually transmitted pathogen with complications like those of *C. trachomatis*, such as pelvic inflammatory disease. These two bacteria not only produce similar symptoms, but 40–46% of individuals infected with *N. gonorrhoeae* are concurrently infected with *C. trachomatis* [22]. Thus, the present findings may have translational relevance for STI management, as the EOs could act against both pathogens without harming human cervical epithelial cells (see Supplementary material).

Our comprehensive chemical characterization of both lemon and pine EOs demonstrated findings consistent with previous reports, involving a mixture of a diverse set of terpenoids and other volatile compounds. Limonene, a monocyclic monoterpene was found in major quantities in both EOs, being a major compound for *C. limon* EO and one of the main components in *P. sylvestris*. Hence, the antichlamydial activity of limonene was confirmed through *C. trachomatis* genome copy and inclusion count quantification as well as infectious progeny production assays. Both enantiomers, S-limonene and R-limonene, were studied using concentrations adjusted to correspond those in the applied lemon EO to evaluate whether the compound contributed to the activity against *C. trachomatis*. Notably, R-limonene (also known as D-limonene), reported in the literature as the enantiomer found in *C. limon* EO [19], exhibited greater antichlamydial activity in our study (Figs. 4 and 5). Even though limonene makes up roughly two thirds of the studied *C. limon* EO, the EO proved to have higher activity against *C. trachomatis* (Fig. 2) than its isolated major constituent (Fig. 4). Although falling beyond the scope of the current study, the various additional compounds found in the EO presumably have potentiating effects which may contribute to the overall activity of the EO. Similarly, while the differences in the antichlamydial activity between lemon and pine EOs could be attributed to the different amount of limonene found, the role of other compounds present in the EO and potential synergies between them cannot be overlooked.

Besides the two EOs included in this study, limonene is found widely in plants such as sweet orange, bitter orange, neroli, mandarin or petitgrain [19]. Limonene is widely used for its citric fragrance as an additive and has been linked to several biological effects, including antioxidant, anti-inflammatory, and cardioprotective activities [23].

As for the possible antibacterial mechanisms of action, many previous studies on limonene against planktonic bacteria have demonstrated its membrane disruption potential. At least in *Escherichia coli*, limonene seems to damage the cell membrane producing alterations in membrane permeability and disrupting the membrane integrity [24]. To assess whether limonene or the EOs could affect *C. trachomatis* EB membrane integrity, viability-PCR assay was performed. According to these data, the EBs treated with EOs and limonene indeed had experienced damage in the EB membrane, indicated by the altered PCR amplification after PMA exposure (Fig. 6). However, despite the effect on the EB membrane permeability, the EB infectivity was not compromised by treatment with the EOs or limonene, as the EB pretreatment did not reduce *C. trachomatis* genome copy numbers after the infection on HeLa-229 cells (Fig. 7). The chlamydial EB form, whose morphology and composition have become increasingly well characterized, is encased by the chlamydial outer membrane complex (COMC). This structure is heavily reinforced by disulfide-cross-linked proteins, including the major Outer Membrane Protein (MOMP), OmcA and OmcB, which confer exceptional rigidity and restrict membrane permeability. Together with surface lipopolysaccharide and additional outer-membrane components, the COMC protects EBs from osmotic and mechanical stress during their extracellular phase [5, 25, 26]. When the EBs are internalized, there is a reorganization of this structure since RBs present reduced disulfide bonds in their COMC proteins, resulting in a less rigid structure essential for their replication and adequate for the stable environment of the inclusion [5, 25]. Hence, the maintained EB infectivity despite lipid membrane alterations may result from unique chlamydial cell wall characteristics and the changes that occur after internalization. In fact, protein components of the EB envelope play a direct role in the internalization process. Several well-characterized surface-exposed and secreted proteins contribute to host cell entry. For instance, OmcB acts as an adhesin that mediates the initial attachment to host cell receptors, while preformed type III secretion system effectors are delivered into the host cell upon contact, triggering actin rearrangements required for uptake. Following internalization, inclusion membrane proteins further remodel the host endomembrane system to promote intracellular survival. Therefore, although limonene and limonene-containing EOs seem to damage the EB lipid membrane, internalization may still occur due to these protein factors [27, 28]. In fact, a recent study on monoclonal antibodies targeting MOMP epitope exposed on the *C. trachomatis* EB surface suggested that despite effectively coating the outer membrane, the antibodies failed to reduce EB infectivity, as they were rapidly lost from the bacterial surface after EB internalization. This illustrates that the membrane remodeling and other changes that EBs undergo after internalization can modify or diminish the functional effects of surface-associated alterations. Regardless of the underlying molecular level interactions, our results indicate that viability-PCR alone may not serve as a reliable method for assessing the infectivity of chlamydial EBs.

Since our data indicated that the impact of the EOs and limonene on EB membrane permeability did not have a significant role in their antichlamydial activity, other mechanisms of action were explored. Anticancer properties of limonene have been widely described, being competitive inhibition of protein prenylation one of the known mechanisms of action [29]. Protein prenylation is a crucial post-translational modification that regulates the localization and function of some membrane-associated proteins in eukaryotes, most notably small GTPases including members of the Ras, Rho, and Rab families. This process involves the covalent attachment of isoprenoid groups, such as farnesyl (C15) or geranylgeranyl (C20) moieties, to conserved cysteine residues at the C-terminus of these proteins [30]. *C. trachomatis* exploits host vesicular trafficking pathways through the recruitment and manipulation of small GTPases, particularly Rab proteins, to facilitate its intracellular survival and replication [6]. However, the inhibition of protein prenylation has never been studied as an antichlamydial strategy. Nevertheless, one earlier study on a closely related species, *Chlamydia pneumoniae,* reported this pathogen to increase small GTPase prenylation in its host cells [31]. In the same study, geranylgeranyl supplementation was found to counteract statin-mediated inhibition of this process. Inspired by these findings, we investigated the involvement of limonene’s competitive inhibition of eukaryotic protein prenylation in its antichlamydial activity. Both farnesyl and geranylgeranyl supplementation of infected cell cultures were found to reverse the inhibitory effect of limonene (Fig. 8). These data suggest that one potential mechanism of action of limonene against *C. trachomatis* is related to the inhibition of protein prenylation through competitive inhibition, since the antichlamydial activity of limonene is lost when the prenyltransferase substrates farnesyl and geranylgeranyl are added to the culture.

In previous literature, limonene has been shown to inhibit protein prenylation, specifically targeting farnesyl transferase (FTase) and geranylgeranyl transferase (GGTase) activity of small GTPases [29, 32]. However, its inhibitory effect is relatively weak, with an IC_50_ exceeding 40 mM (5449.6 µg mL^−1^) when assayed on enzymes isolated from rat brain cytosol. Given the high IC_50_, it is evident that relatively high limonene concentrations are required to achieve meaningful effects on cellular protein prenylation patterns. While the concentrations applied in our current work to achieve chlamydial inhibition were relatively high, they are still lower than the above-mentioned value for the inhibition of isolated FTase and GGTase. Another earlier study reported that limonene inhibits isoprenylation in *Plasmodium falciparum*, a process essential for the function of its Ras- and Rap-like proteins, with an IC_50_ value of 1.22 mM (166.2 µg mL^−1^), [33, 34]. The discrepancies in IC₅₀ values between the previously mentioned study, which used enzymes extracted from rat brain cytosol, and the results obtained in cell-based models are particularly noteworthy. Since previous reports have shown that limonene metabolites exhibit greater potency as prenylation inhibitors than limonene itself [32, 35], these differences may reflect some degree of modification or metabolic transformation occurring within the cellular environment. One previous study reported that, although metabolic transformations of limonene have been observed in vivo, no such metabolism was detected in cell culture [36]. However, this result was obtained on a 3 h incubation in a mouse embryonic fibroblast cell line, leaving open the possibility that cell types more competent in xenobiotic metabolism or longer incubation times, such as the 48-h used in our experiment, could have different outcomes.

To elucidate whether limonene metabolites displayed antichlamydial activity, the impact of perillic acid and perillyl alcohol was evaluated (Fig. 8). Perillyl alcohol is the major metabolite of limonene that naturally occurs in the essential oils of several plants, including mint, lavender, lemongrass, and sage. In human plasma, one of the main metabolites derived from both limonene and perillyl alcohol is perillic acid [29]. According to our data, perillyl alcohol reduced the number of *C. trachomatis* inclusions at concentrations significantly lower than those tested for limonene. Besides, as shown in Fig. 8, the antichlamydial activity of perillyl alcohol was also impaired by the addition of farnesyl and geranylgeranyl. These results indicate that perillyl alcohol displays higher antichlamydial potency than limonene and is also related to the inhibition of protein prenylation. On the other hand, perillic acid did not show antichlamydial activity (data not shown). Duelund et al. [37] demonstrated that perillic acid shows virtually no partitioning into the lipid bilayer, indicating that it does not significantly interact with the hydrophobic core of biological membranes [37]. Therefore, it is possible that this compound is not internalized to eukaryotic cells and consequently lacks antichlamydial activity, despite previous reports suggesting its inhibitory activity on protein prenylation [32, 38].

The herein suggested host–pathogen interface-targeting approach (disrupting host isoprenoid-dependent processes) that limonene and its metabolite perillyl alcohol possess, represents a previously undescribed pharmacological approach for tackling chlamydial infections. Our findings suggest that future studies on protein prenyltransferase inhibitors or other small molecules targeting cellular isoprenoid synthesis may pave the wave for drug repurposing to treat intracellular bacterial infections. These results hence emphasize the relevance of natural products in the identification of novel targets and mechanisms of action for the treatment of infectious diseases.

## Supplementary Information

Supplementary material 1

Supplementary material 2

## Data Availability

All data are available from the authors upon request.
