# Supplementary material for: Limonene and its metabolite perillyl alcohol inhibit Chlamydia trachomatis growth by altering host isoprenoid metabolism
Source: Nat Prod Bioprospect. 2026 Apr 13;16(1):51. doi: 10.1007/s13659-026-00611-5 (PMC13070884; doi:10.1007/s13659-026-00611-5)
Supplement: Supplementary file 1 — Supplementary material 1 [file 13659_2026_611_MOESM1_ESM.docx]

Natural Products and Bioprospecting

Limonene and its metabolite perillyl alcohol inhibit Chlamydia trachomatis growth by altering host isoprenoid metabolism

Pilar Cebollada^a^, Inés Reigada^b†^, Maarit Ylätalo^b†^, Candela Gerediaga^a^, Víctor López^a,c^ Leena Hanski ^b*^

*^a^Department of Pharmacy, Faculty of Health Sciences, Universidad San Jorge, 50830 Villanueva de Gállego (Zaragoza), Spain.*

*^b^Drug Research Program, Division of Pharmaceutical Biosciences, Faculty of Pharmacy, University of Helsinki, 00014, Helsinki, Finland.*

*^c^Instituto Agroalimentario de Aragón-IA2, CITA-Universidad de Zaragoza, 50013 Zaragoza, Spain*

^†^ These authors contributed equally to this work

^*^**Corresponding author:**

Leena Hanski, Drug Research Program, Division of Pharmaceutical Biosciences, Faculty of Pharmacy, University of Helsinki, 00014, Helsinki, Finland.

Tel.: +358 29 415 9164

E-mail address: [leena.hanski@helsinki.fi](mailto:leena.hanski@helsinki.fi)

Online resource 1 includes the results of cell viability assays performed on HeLa cells.

Cells were seeded in 96-well plates (300 000 cells/ml) and incubated for 24 h before treatment with various EO/pure compound concentrations in µg ml ^-1^. After 48 h, cells were washed with PBS, stained with 20 µM resazurin, and incubated for 2 h. Fluorescence (λ_ex_ = 560 nm, λ_em_ = 590 nm) was measured using a Varioskan LUX microplate reader (Thermo Scientific, Waltham, MA, US).

**Table S.1** Cell viability values of HeLa cells exposed to both EOs at different concentrations for 48 h. Cell viability percentages of HeLa cells exposed to *C. limon* and *P. sylvestris* for 48 h.

| EO | 31.25 | 62.5 | 125 | 250 | 500 | 1000 | 2000 |
| --- | --- | --- | --- | --- | --- | --- | --- |
| C. limon | 92.02 | 87.65 | 86.08 | 90.08 | 89.43 | 93.17 | 97.55 |
| P. sylvestris | 99.25 | 98.48 | 96.96 | 94.72 | 93.63 | 96.90 | 98.87 |

**Table S.2** Cell viability percentages of HeLa cells exposed to limonene. Cell viability percentages of HeLa cells exposed to limonene S (-) and limonene R (+) for 48 h.

| EO | 175 | 262.5 | 350 | 525 | 700 | 1050 | 1400 |
| --- | --- | --- | --- | --- | --- | --- | --- |
| Limonene S (-) | 87.69 | 89.02 | 85.91 | 95.68 | 96.20 | 66.45 | 30.72 |
| Limonene R (+) | 95.94 | 97.83 | 97.33 | 97.86 | 97.81 | 98.11 | 100.50 |

**Table S.3** Cell viability values of HeLa cells exposed to limonene metabolites. Cell viability percentages of HeLa cells exposed to perillyl alcohol and perillic acid at different concentrations for 48 h.

|  | 50 | 100 | 250 | 500 |
| --- | --- | --- | --- | --- |
| Perillyl alcohol | 114.24 | 86.03 | 7.80 | 7.61 |
| Perillic acid | 92.24 | 92.91 | 84.71 | 57.49 |
