# Supplementary material for: Limonene and its metabolite perillyl alcohol inhibit Chlamydia trachomatis growth by altering host isoprenoid metabolism
Source: Nat Prod Bioprospect. 2026 Apr 13;16(1):51. doi: 10.1007/s13659-026-00611-5 (PMC13070884; doi:10.1007/s13659-026-00611-5)
Supplement: Supplementary file 2 — Supplementary material 2 [file 13659_2026_611_MOESM2_ESM.docx]

Natural Products and Bioprospecting

Limonene and its metabolite perillyl alcohol inhibit Chlamydia trachomatis growth by altering host isoprenoid metabolism

Pilar Cebollada^a^, Inés Reigada^b†^, Maarit Ylätalo^b†^, Candela Gerediaga^a^, Víctor López^a,c^ Leena Hanski ^b*^

*^a^Department of Pharmacy, Faculty of Health Sciences, Universidad San Jorge, 50830 Villanueva de Gállego (Zaragoza), Spain.*

*^b^Drug Research Program, Division of Pharmaceutical Biosciences, Faculty of Pharmacy, University of Helsinki, 00014, Helsinki, Finland.*

*^c^Instituto Agroalimentario de Aragón-IA2, CITA-Universidad de Zaragoza, 50013 Zaragoza, Spain*

^†^ These authors contributed equally to this work

^*^**Corresponding author:**

Leena Hanski, Drug Research Program, Division of Pharmaceutical Biosciences, Faculty of Pharmacy, University of Helsinki, 00014, Helsinki, Finland.

Tel.: +358 29 415 9164

E-mail address: [leena.hanski@helsinki.fi](mailto:leena.hanski@helsinki.fi)

Online resource 2 includes the chemical structures of the major constituents identified in the essential oils.

*Chemical structures*

**Figure S.1** Chemical structures of the main compounds found in *C. limon* EO.


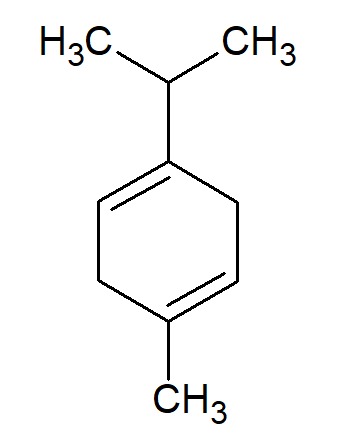


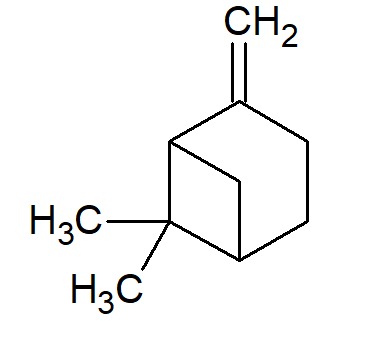

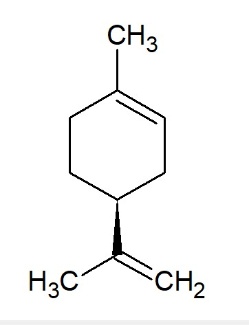


**Figure S.2** Chemical structures of the main compounds found in *P. sylvestris* EO.


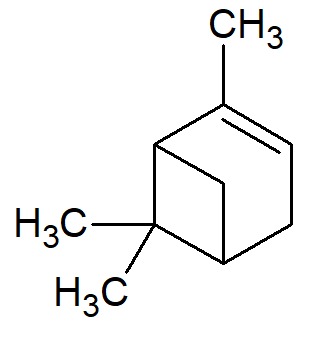

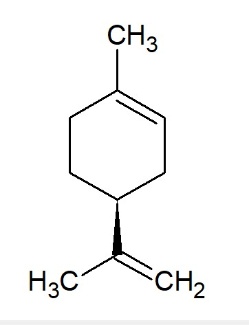

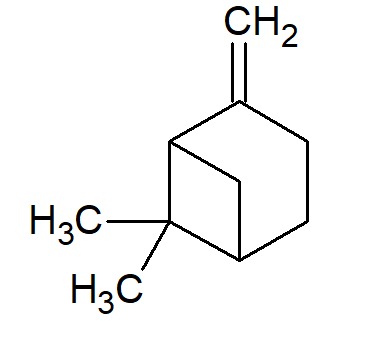


**Figure S.3** Chemical structures of limonene, perilic acid and pery.
